# Natural Antimicrobial Compounds as Promising Preservatives: A Look at an Old Problem from New Perspectives

**DOI:** 10.3390/molecules29245830

**Published:** 2024-12-10

**Authors:** Ludmila Yarmolinsky, Faina Nakonechny, Tigabu Haddis, Boris Khalfin, Arik Dahan, Shimon Ben-Shabat

**Affiliations:** 1 Faculty of Health Sciences, Ben-Gurion University of the Negev, Beer-Sheva 8410501, Israel; yludmila@post.bgu.ac.il (L.Y.); boriskh83@gmail.com (B.K.); 2 Department of Chemical Engineering, Ariel University, Ariel 4070000, Israel; fainan@ariel.ac.il (F.N.); tigabu@ariel.ac.il (T.H.)

**Keywords:** phytochemicals, photosensitizers, natural products, food safety, food conservation

## Abstract

Antimicrobial compounds of natural origin are of interest because of the large number of reports regarding the harmfulness of food preservatives. These natural products can be derived from plants, animal sources, microorganisms, algae, or mushrooms. The aim of this review is to consider known antimicrobials of natural origin and the mechanisms of their action, antimicrobial photodynamic technology, and ultrasound for disinfection. Plant extracts and their active compounds, chitosan and chitosan oligosaccharide, bioactive peptides, and essential oils are highly potent preservatives. It has been experimentally proven that they possess strong antibacterial capabilities against bacteria, yeast, and fungi, indicating the possibility of their use in the future to create preservatives for the pharmaceutical, agricultural, and food industries.

## 1. Introduction

Losses of food products (approximately 1.3 billion tons every year) occur because of decay and spoilage worldwide [1]. This problem requires increased attention not only because of the loss of valuable products but also because these losses harm the environment [2]. *Clostridium botulinum*, *Bacillus cereus*, *Staphylococcus aureus*, *Salmonella*, *Campylobacter*, *Escherichia coli*, *Listeria monocytogenes*, *Vibrio cholerae,* and other pathogenic bacteria are widespread in many kinds of food; they may cause food-borne diseases [3].

For instance, only in the USA, more than 1 million people become infected with *Salmonella* every year, resulting in 19,000 hospitalizations and 380 deaths [4]. Another example is *Clostridium botulinum*. This microbe can contaminate canned fish, meat products, vegetables, and mushrooms; produce botulinum toxin, and cause the fatal disease botulism [5].

A recent understanding of this problem suggests the use of various preservation techniques (cold storage, improved packaging, ionization, etc.) and food preservatives [6]. Preservatives are compounds that can maintain current conditions, increase the shelf life of products, and prevent damage from oxidation, temperature, light, and microorganisms. Microorganisms are the most important causes of damage. Hence, the agents used for preservation should have effective antimicrobial properties. Well-known food preservatives (sodium benzoate; acetic, lactic, benzoic, and sorbic acids; hydrogen peroxide; and chelators) are approved by the Food and Drug Administration (FDA) because they inhibit the growth of bacteria, yeast, and mold [7,8] and comply with the strict requirements of the food industry [9]. According to the World Health Organization, not more than 5 mg/kg of benzyl alcohol, benzoic acid and sodium benzoate may be permitted [10]. Overall, evidence for their adverse health effects is known, and for this reason, their use in many food products has been heavily restricted. For example, various adverse effects of sodium benzoate have been reported, including a negative influence on hormones and fertility and the ability to cause oxidative stress and mutagenic effects [11]. In addition, many of the preservatives in pharmaceutical and cosmetic products are not safe [12]. For example, parabens are the most used preservatives in various cosmetic products because of their cheapness and antimicrobial properties, but there is experimental proof of their abilities to trigger mitochondrial dysfunction, oxidative stress in cells [13], and immunological disorders [14].

From this perspective, the application of various natural compounds may hold great promise for identifying less toxic and more effective preservatives than widespread agents [15]. Toxicological studies on many natural products have demonstrated the absence of any adverse effects, even at high doses. Although the high efficacy and low toxicity of such products are well known, they are not widely used in industry because of insufficient technological studies, the complexity of production, standardization problems, and strict industrial requirements in many aspects [15,16,17].

Natural preservatives can be obtained from plants, animal sources, microorganisms, algae, or mushrooms. Moreover, natural objects contain substances that not only have antimicrobial properties [15,18,19,20] but also produce health benefits due to their various medicinal features, including antiviral [19,21], anti-inflammatory [22,23,24], anticarcinogenic [23,25], antidiabetic [23,24,25,26], antifatigue [27], antioxidant [28], antihypertensive [29], antihyperlipidemic [30], cardioprotective [31], hepatoprotective [32], nephroprotective [33], and wound healing [18,20] effects. However, few of these agents are used on an industrial scale.

Although the antimicrobial properties of natural products have been described in numerous reviews [15,34,35,36,37,38,39,40], many aspects of food and drug disinfection have not yet been presented in full detail. Considering the unavailability of effective natural products for industry, it seems appropriate to review these agents and their properties. A comprehensive search of electronic databases (PubMed, Google Scholar, Scopus, and Science Direct) since 1998 was performed. The multiple criteria sorting method was used [41].

Detailed knowledge of chemical composition, biological properties, safety profile, and environmental toxicity is essential for the development of novel natural preservatives. The aim of this review is to critically evaluate various antimicrobials of natural origin and their mechanisms of action, antimicrobial photodynamic technology, and ultrasound for disinfection.

## 2. Antimicrobial Compounds of Natural Origin and Their Mechanisms of Action

According to the literature, polyphenols, terpenoids, sulfides, coumarins, saponins, furils, alkaloids, polyines, thiophenes, different sugars, fatty oils, resins, glycosinolates, proteins, and peptides have antimicrobial properties [15].

Polyphenols constitute the largest group of antimicrobial compounds (more than 8000 phenolic structures), which includes phenolic acids, flavonoids, lignans, stilbenes, amides, etc. [34]. Although the exact antimicrobial modes of action of many compounds are not yet fully understood, they have diverse sites of action at the cellular level. As shown in Table 1, the most widespread mechanism of action involves disrupting the structure of the bacterial cell membrane. The mechanisms of antimicrobial action of pure compounds isolated from natural products are presented in Table 1.

The results of an electronic search of several databases (PubMed, Google Scholar, Scopus, and Science Direct) since 1998 demonstrated that the isolation and identification of antimicrobial compounds from many selected plant, animal, microorganism, algae, or mushroom sources have not been completed, but even if antimicrobial compounds were identified, the mechanism of their antimicrobial action remained unknown in many cases. Understanding the mechanisms of their antimicrobial activities is vital for their rational use in medicine and industry.

An interesting example is the synergism between resveratrol (a natural phenolic stilbene) and aminoglycosides and cationic antimicrobial peptide antibiotics. The activity of resveratrol against bacteria is relatively low [36,37]. The combination of resveratrol with the above-mentioned antibiotics was significantly effective [38].

The combinations of several essential oils with conventional antimicrobial agents showed strong synergistic activity in many cases [42,43,44]. In addition, the enhanced antimicrobial activities of several essential oil combinations were reported [45,46]. It was shown that allicin, a volatile compound extracted from raw garlic with antimicrobial properties, may be more effective in combination with other antimicrobials than when it functioned alone [47].

Importantly, some compounds of natural origin are not bactericidal, but they are effective in combination with antibiotics. For example, skyllamycins B and C are cyclic depsipeptides of natural origin that increase the therapeutic efficacy of azithromycin [39]. These antibiotics are not effective in the presence of biofilms, whereas skyllamycins B and C can inhibit biofilm formation, thereby increasing the effectiveness of the antibiotics [39].

**Figure 1:**
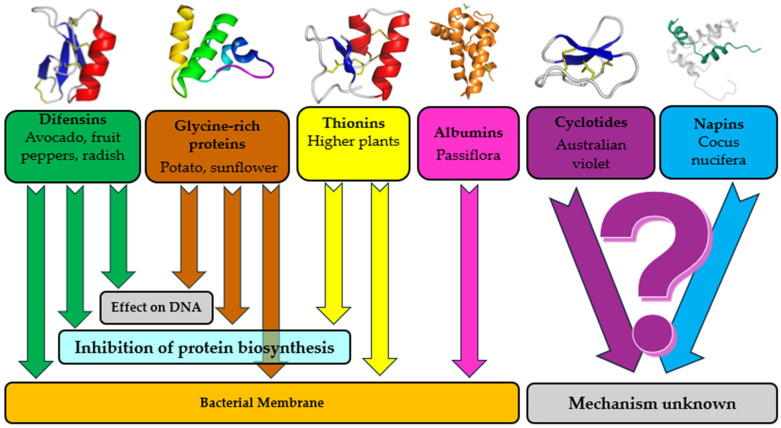
The plant-derived antimicrobial peptides (AMPs) and mechanisms of their antibacterial and antifungal activities.

Plant-derived antimicrobial peptides (AMPs) represent a very interesting and promising class of compounds. They include several important groups with antibacterial and antifungal properties: defensins, albumins, glycine-rich proteins, thionins, cyclotides, and napins (Figure 1) [40,48].

Figure 1 shows the basic antibacterial and antifungal mechanisms of AMPs [49]. AMPs are known to disrupt bacterial membranes or demonstrate nonmembrane target mechanisms [50], which include the inhibition of protein biosynthesis, protease activity, nucleic acid biosynthesis [51], the production of reactive oxygen species (ROS) [52], and the inhibition of cell division [53].

Although the membrane target mechanisms are largely unknown, several hypotheses are associated with the activities of AMPs, such as the carpet model, electroporation, membrane thinning or thickening, nonlytic membrane depolarization, pore formation, oxidized lipid targeting, barrel stave, and nonbilayer intermediate [54].

Although AMPs are promising antimicrobial agents, they are not used in industry. In fact, these compounds have not yet been researched in depth; in many cases, the mechanism of their antimicrobial activity is not known. The main drawbacks of natural plant AMPs include poor chemical stability, short-term effectiveness, and toxicity [55].

**Table 1:** Natural antibacterial compounds.

Compounds	Origin	Mechanism of Action	References
Allicin	Garlic	Destruction of the synthesis of DNA, RNA, and some proteins	[56]
Aloe-emodin	*Aloe vera*	Inhibition of biofilm development and extracellular protein production	[57]
Buforin II	Orinoco lime treefrog (*Sphaenorhynchus lacteus*)	Membrane disruption	[58]
Caffeic acid	Herbs of the mint family, sunflower seeds, apricots, prunes, coffee beans	Inhibition of RNA polymerase	[59]
Cecropin A	Silk moth	Membrane disruption	[60]
Chitosan	Crustacean shells, fungi and algae cell walls	Electrostatic interactions occur between cationic chitosan and anionic molecules at the microbial cell surface, which may lead to cell wall disruption and intracellular component leakage; can penetrate the cell membrane and interact with DNA, thereby interfering with protein synthesis processes	[61]
Chlorogenic acid	Eggplants, prunes, peaches, apples, coffee beans	Membrane disruption	[62]
Citral	Essential oils of many plants	Membrane disruption	[63]
Daidzein	Soybeans and other legumes	Inhibition of DNA topoisomerases	[64]
Divaricatic acid	Lichen, *Evernia mesomorpha*	Inhibition of nucleotide synthesis	[65]
Epicatechin 3-gallate	Green tea	Membrane destruction	[66]
Epigallocatechin-3-gallate	Tea	Inhibition of efflux pumps	[67]
Eugenol	Essential oils of many plants	Membrane disruption	[68]
Genistein	Some plants	Inhibition of DNA topoisomerases	[69]
Geraniol	Essential oils of many plants	Destruction of cell wall function by downregulating the activity of plasma membrane ATPase and reducing ergosterol levels	[70]
Glabrol	*Glycyrrhiza* species	Membrane destruction	[71]
Kaempferol	Plants	Membrane disruption	[72]
Lactobionic acid	Caspian Sea yogurt	Induction of oxidative stress, loss of membrane integrity, and inhibition of metabolic pathways, protein synthesis, and DNA repair. In addition, in Gram-negative bacteria, an increase in the permeability of the outer membrane that causes hypoosmotic shock was observed.	[73,74]
Licochalcone	*Glycyrrhiza inflata*	Inhibition of NADH-cytochrome c reductase	[75]
Linalool	Many flowers, spice plants	Membrane disruption	[76]
Luteolin	Many herbs of the mint family, celery, broccoli, green pepper, carrots, olive oil	Membrane disruption	[77]
Magainin	African clawed frog	Membrane disruption, interfering with cell metabolism, and targeting different cytoplasmic components	[78]
Mellitin	Bee venom	Membrane disruption	[79]
Morin	*Maclura pomifera*, *Maclura tinctoria*, *Psidium guajava*	Promotion of bacterial aggregation, intervention in the biofilm growth, suppression of the PBP2a-mediated resistant mechanism of action, and membrane disruption	[80]
Myricetin	Vegetables, fruits, nuts, berries, tea, red wine	Inhibition of the activity of hemolysin and p38	[81]
P-coumaric acid	Peanuts, navy beans, tomatoes, carrots, basil, garlic	Increasing the membrane permeability, binding to the phosphate anion of DNA.	[82]
Polyphemusin	American horseshoe crab, *Limulus Polyphemus*	Membrane disruption	[83]
Protegrins	Porcine leukocytes	Membrane disruption	[84]
Quercetin	Honey, plants	Membrane disruption, change in membrane permeability, inhibition of synthesis of nucleic acids and proteins, reduction in the expression of virulence factors, mitochondrial dysfunction, and preventing biofilm formation, inhibition of quorum sensing.	[69,85]
Resveratrol	Several plants	Suppression of FtsZ expression, ATP synthase activity inhibition	[86]
Rhodomyrtosone B	*Rhodomyrtus tomentosa*	Membrane disruption	[87]
Trans-cinnamaldehyde	Cinnamon	Membrane disruption	[88]

## 3. Natural Compounds in Antimicrobial Photodynamic Therapy

**Figure 2:**
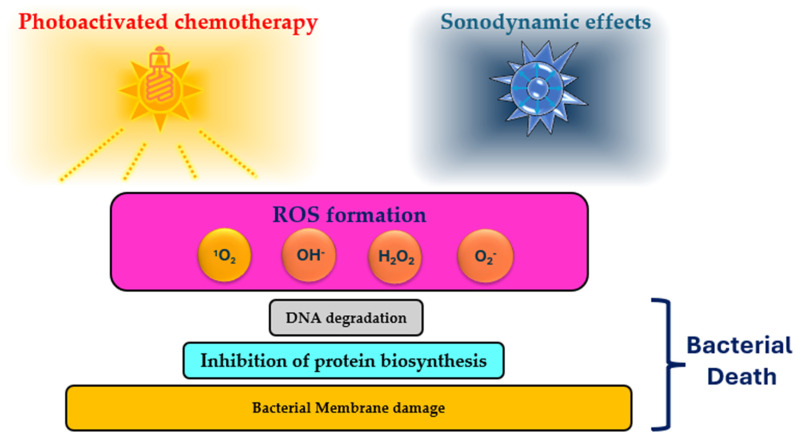
Bacterial cell death after photodynamic or sonodynamic treatments.

Antimicrobial photodynamic therapy is a light-based method to inactivate microorganisms [89]. This technology is also referred to in the literature as photodynamic therapy (PDT), photoactivated chemotherapy (PACT), photodynamic disinfection (PDD), light-activated disinfection (LAD), and photoactivated disinfection (PAD) [90]. Light has been recognized for its ability to treat various conditions since ancient times. However, significant advancements in this field began in 1960 after Macmillan reported that toluidine blue effectively countered microorganisms within 30 min of irradiation with 21–30 mW of light at 632 nm [91]. Other compounds, such as methylene blue, rose bengal, eosin Y, neutral red, acridine orange, crystal violet, and rhodamine 6G, possess similar antimicrobial properties when activated by light (Figure 2). These compounds were determined to be photosensitizers (PSs) [89]. Photodynamic antimicrobial agents primarily elicit their antimicrobial effects by generating ROS upon light activation. When exposed to light, the excited photosensitizer transfers energy to molecular oxygen, resulting in the production of ROS such as singlet oxygen, superoxide radicals, and hydroxyl radicals [92]. These ROS harm microbial structures, affecting lipids, proteins, and nucleic acids, leading to cell death [89,92]. Additionally, photodynamic antimicrobial agents can target microbial membranes, compromising their integrity, causing leakage of cellular components, and ultimately resulting in microbial inactivation [92,93].

In recent years, there has been interest in using natural compounds to develop photodynamic antimicrobial agents. These compounds, which are derived from various natural sources, offer a sustainable and environmentally friendly alternative to conventional antimicrobial agents [94,95]. The utilization of natural sources for PDT provides a rich pool of compounds with diverse chemical structures and properties. Natural compounds such as porphyrins, chlorophylls, curcumin, and phthalocyanines have shown promising antimicrobial activity when activated by light at appropriate wavelengths (Table 2). At present, more than 100 PSs of natural origin are known [96].

Photosensitizers exhibit broad-spectrum antimicrobial activity and are capable of targeting bacteria [97,98], fungi [99,100], viruses [89], and even antibiotic-resistant strains [90]. This versatility makes them valuable for preserving a wide range of drugs and food products. These compounds, which are derived from natural sources, offer a natural and eco-friendly alternative to synthetic preservatives. They are generally considered safe for consumption, reducing concerns about potential health risks associated with synthetic preservatives [91]. Antimicrobial photosensitizers have been shown to effectively extend the shelf life of drugs and food products by inhibiting microbial growth and spoilage [92]. Moreover, these compounds have demonstrated the ability to penetrate and disrupt biofilms, effectively eliminating biofilm-associated pathogens and enhancing preservation efficacy [93]. All this can have significant economic benefits by reducing product waste and ensuring product quality during storage and transportation.

Further research and optimization are required to harness the full potential of these natural photodynamic antimicrobials for clinical applications, paving the way for innovative and sustainable antimicrobial strategies.

**Table 2:** Natural antimicrobial compounds used in photodynamic therapy (PDT).

Compounds	Origin	Microorganism	Mechanisms of Action	Treatment Parameters	Effect	References
Aloe-emodin (AE)	*Aloe vera*	*Pseudomonas aeruginosa*	Light irradiation triggers ROS generation, causing damage to bacterial cells and disrupting their structure and function	Wavelength: 435 ± 10 nm, 80 Mw/cm^2^, AE concentration: 0.5–100 μM for 10–40 min	AE concentration and light energy dose-dependent inactivation	[101]
		*Staphylococcus aureus* biofilm	Disruption of membrane unity, increasing cell membrane permeability	Wavelength: 450 and 460 nm, 40 mW/cm^2^, AE concentration: 512 µg/mL for 10 min	Nucleic acid and protein release	[102]
Caffeic acid (CA)	Natural polyphenol fruits and vegetables (sunflower seeds, apricots, prunes, coffee beans)	*E. coli*, *Salmonella enterica serovar typhimurium,* and *Listeria monocytogenes*	Inhibiting bacterial enzyme activity, including respiratory enzymes, and damaging the inner cell structure by producing ROS within the cells	Wavelength: 400 nm, light doses: 3, 4, and 5 J/cm^2^, CA concentration: 3 mM	Considerable damage, such as compromised cell membranes and disrupted intracellular structures, resulted in a decrease in all three pathogens.	[103]
*Chlorella* and *Curcuma* extracts	Chlorella	*Streptococcus mutans* (*S. mutans*)	PDT harmed biofilm bacteria by disrupting their cellular structure through ROS generated from the interaction of natural extracts with the biofilm	Wavelength: 405 nm, 17.7 J, extract concentration: 0.5 mg/mL for 5 min	Reduction in viable cells in the biofilm by 11% and 25%, respectively, compared to the control biofilm	[104]
Chlorophyll derivatives	Green pigment found in plants (spinach, parsley, alfalfa); algae, cyanobacteria	*S. aureus*, *S. mutans*, *P. acnes*, *E. coli*, *Candida albicans*	ROS generation, membrane disruption, cellular component damage, and oxidative stress induction	Wavelength: 700–800 nm, power density: 30 mW/sm^2^, light dose density: 36 J/sm^2^, concentration: 5 µM, average time: 20 min	Strong antimicrobial activity against *S. aureus*, *E. coli*, and *Candida albicans* via ROS generation, membrane disruption, and cellular damage.	[105]
Curcumin	*Curcuma longa*	*S. aureus*, *E. coli*, *L. monocytogenes*	Producing ROS, disrupting membrane unity, increasing cell membrane permeability	Wavelength: 470 nm, PS concentration: 2.5 µM, irradiation intensity: 60 mW/cm^2^, incubation period: 30 min	Physiological and biochemical changes and damage to bacterial cell components, including DNA, proteins, and lipids, ultimately result in cell death.	[106]
Myricetin	Vegetables, fruits, nuts, berries, tea, red wine	*Streptococcus mutans* and *Streptococcus sobrinus*	Oxidative damage to cell membranes and intracellular components like cytoplasmic proteins and DNA	Irradiation: 200 mM/cm^2^, concentration: 12.5%, 5 min	PDT using 0.8% pomegranate and 3% chokeberry juice damaged approximately 5log_10_ of *S. sobrinus* and *S. mutans*. Bilberry juice at 12.5% concentration affected both strains. Pomegranate at 25% and bilberry and chokeberry at >50% reduced mixed bacteria.	[107]
Polyphenols	*Rumex cristatus* DC, *Cotinus coggygria* Scop, *Beta vulgaris* L. var. *cicla*, and *Eruca sativa*	*Streptococcus mutans*	Generating ROS to destroy bacteria by damaging their cell walls, membrane proteins, and nucleic acids	PSs concentration: 0.23–0.41 g/mL, wavelength: 600 nm	Reducing microorganisms by up to 99%	[108]
Quercetin (QCT)	Honey, onions, grapes, berries, cherries, broccoli, and citrus fruits	*Streptococcus mutans*	Membrane disruption, change in membrane permeability, inhibition of the synthesis of nucleic acids and proteins, reduction in the expression of virulence factors, mitochondrial dysfunction, prevention of biofilm formation, inhibition of quorum sensing	Wavelength: 405 nm, intensity: 150 mW/cm^2^, 60 s	The MBIC of QCT against *S. mutans* was 128 µg/mL. Significant degradation was observed in biofilms treated with PDT relative to the control group.	[109]
		*E. coli* and *L. monocytogenes*	ROS generation causes membrane damage to bacterial cells, resulting in their inactivation (type I dominant mechanism)	Blue LED light at 405 nm, 17–102 min	The combination treatment of quercetin-mediated antimicrobial PDT with blue light resulted in an additional maximum reduction of 3.01 log for *E. coli* and 5.52 log for *L. monocytogenes* compared to blue light treatment alone.	[110]
Resveratrol	Grapes, berries, peanuts, pines	*Staphylococcus aureus*	Generation of singlet oxygen, which exhibits antimicrobial activity	Wavelength: 660 nm, Power density: 75 mW/cm^2^, concentration: 2 mg/mL, 5 min	Increased antibacterial activity against *S. aureus*, singlet oxygen generation contributing to antimicrobial effects, reduced bacterial load and inflammation in vivo, enhanced production of cytokines TNF-α and IL-17A.	[111]

Photodynamic therapy (PDT) involves the use of photosensitizing agents (PS) activated by specific light wavelengths to induce localized cell damage, particularly in microbial cells. In the context of antimicrobial therapy, PDT offers a promising alternative to traditional antibiotics by targeting a broad spectrum of microorganisms, including bacteria, fungi, and viruses, while minimizing the development of antibiotic resistance. The mechanism of action typically involves the generation of ROS upon light activation of the PS, leading to oxidative damage to microbial cell membranes, proteins, and nucleic acids, ultimately resulting in cell death or inactivation [94,97]. Various natural compounds have been investigated as PSs in antimicrobial PDT. These natural compounds have shown promising antimicrobial properties, making them attractive candidates for use in PDT-based antimicrobial therapy. However, further research is needed to optimize their efficacy, elucidate their mechanisms of action, and evaluate their safety and clinical applicability. Overall, PDT represents a versatile and potentially effective approach for combating microbial infections, particularly in cases of antibiotic-resistant pathogens, while offering the advantages of specificity, minimal side effects, and a reduced likelihood of resistance development [94,95,96].

## 4. Natural Sonodynamic Antimicrobials

Natural sonodynamic antimicrobials, a burgeoning area of research, show great potential in combatting microbial infections via the use of natural compounds activated by ultrasonic waves. These compounds, derived from sources such as plant extracts, utilize the power of sonodynamic therapy (SDT) to eradicate pathogenic microorganisms effectively [112].

SDT is conceptually akin to PDT, but instead of light, ultrasound is employed to activate the sensitizer, generating reactive species that are toxic to microbes. Figure 2 shows a schematic representation of ultrasound-mediated cell damage during SDT. Ultrasound energy can be focused precisely on a specific treatment area with minimal impact on surrounding healthy cells. Moreover, sonosensitizers have low toxicity and exhibit bioactivity only under the influence of ultrasonic activation. Additionally, ultrasound has greater tissue penetration than light does, which influences deep infections [113]. Low-intensity ultrasound can also disrupt the cell membrane, increasing its permeability to sonosensitizers [112,114].

ROS, such as singlet oxygen, hydroxyl radicals, and superoxide anions, play crucial roles in the antimicrobial effects of sonodynamic therapy. Although the exact mechanism of SDT remains unknown, it may involve ultrasonic cavitation, sonochemical effects, and ultrasound-induced apoptosis [115,116]. The type of sonosensitizer, biological system parameters, and ultrasound characteristics significantly influence the mechanism of SDT [114,115,116].

While the antibacterial activity of synthetic photosensitizers has been extensively studied, natural sonodynamic antimicrobial agents are less studied. Among natural sensitizers, curcumin (from *Curcuma longa*) has shown promise, effectively inactivating the foodborne bacteria *B. cereus* [117], *E. coli* [117,118], and *Staphylococcus aureus* [118] under ultrasonic treatment. Another natural compound with sonodynamic properties, hypocrellin B (from *Hypocrella bambuase*), exhibited significant antibacterial effects on methicillin-resistant *Staphylococcus aureus* (MRSA) [119], disrupting membrane integrity without damaging bacteria [120].

Furthermore, natural sonodynamic antimicrobials have demonstrated promising antibiofilm activity by inhibiting biofilm formation, reducing the amount of extracellular polymeric substances, and increasing the susceptibility of biofilm-embedded microorganisms to SDT [112]. Table 3 lists examples of natural antimicrobial compounds used in sonodynamic therapy.

Potential clinical applications of natural sonodynamic antimicrobials include wound healing [121], dermatological infections [122], oral diseases [123], and systemic infections [122]. By harnessing the power of nature and SDT, these compounds offer a sustainable and effective therapeutic approach [112,115,116].

However, further research is necessary to fully understand their potential and address existing challenges. Standardizing extraction methods, optimizing treatment parameters, and understanding interactions with host cells are among the challenges and limitations associated with natural sonodynamic antimicrobial agents. Future research directions include developing new natural compounds and advanced delivery systems.

**Table 3:** Natural antimicrobial compounds used in SDT.

Compounds	Origin	Microorganism	Ultrasound Parameters	Effect	References
Curcumin	*Curcuma longa*	*Streptococcus mutans*	Frequency: 1 MHz, pulse repetition frequency: 100 Hz, ultrasonic intensity: 1.56 W/cm^2^, curcumin concentration: 50 mM for 1 min	ROS was excessively generated after Cur and NM@Cur-mediated SDT, possibly responsible for antimicrobial effects	[124]
		*Listeria monocytogenes*	Wavelength: 490 and 520 nm, curcumin concentration: 3.7 mg/mL, ultrasound treatment: 600 and 800 W for 25 and 30 min	ROS-induced damage of cell membranes, DNA, and proteins	[125]
		*Staphylococcus aureus* biofilm	Frequency: 1 MHz, power density: 3 W/cm^2^, duty cycle: 20%, pulse frequency: 100 Hz for a duration of 15 min	ROS (mostly hydroxyl radicals) production, reduction in cellular metabolism	[122]
		*Staphylococcus aureus* biofilm	Frequency: 1 MHz, power density: 3 W/cm^2^, duty cycle: 20%, pulse frequency: 100 Hz	Reduction in the adhesion ability of the bacteria, reduction in cell metabolism, change in biofilm morphology characteristics	[126]
		*Bacillus cereus* and *Escherichia coli*	Frequency: 1 MHz, intensity (*ISATA*): 1.56 W/cm^2^, 35 min	Production of ROS through the interaction of ultrasound, sonosensitizer, and molecular oxygen	[117]
Curcumin (CUR) and Tanshinone IIA (TSIIA)	*Curcuma longa* (CUR) and *Salvia miltiorrhiza* (TSIIA)	*Staphylococcus aureus*	Concentration: 12.5 µg/mL, frequency: 1 MHz, sound intensity output: 3 W/cm^2^ for 10 min		[127]
Nanocurcumin	*Curcuma longa*	*Enterococcus faecalis* and *Candida albicans* biofilm	Power: 3 W/cm^2^, frequency: 1 MHz, 1 min	The thickness of biofilm significantly decreases due to an increase in the level of ROS	[128]
Nanomicelle curcumin (NM@Cur)	*Curcuma longa* L.	*Acinetobacter baumannii*	Ultrasound power outputs of 28.7, 36.9, and 45.2 mW/cm^2^	Regulation of gene expression involved in the pathogenesis of *A. baumannii*	[129]
Nanoemodin (1,2,8-trihydroxy-6-methylanthraquinone)	Rhubarb	Multi-species bacterial biofilms containing *Staphylococcus aureus*, *Pseudomonas aeruginosa*, and *Acinetobacter baumannii*	5 min, frequency: 1 MHz, pulse repetition frequency: 100 Hz, spatial average ultrasonic intensity: 2 W/cm^2^	Significant reduction in gene expression levels of *lasI*, *agrA*, and *abaI* on multi-species bacterial biofilms	[130]
Hypericin nanoparticles	*Hypericum perforatum*	*S. mutans* biofilms	Frequency: 30 KHz, pulse repetition frequency: 100 Hz, 60 s	Production of ROS, downregulation of biofilm-associated genes (*gtfD*, *comDE*, and *smuT*), and suppressing expression of genes associated with persister cell formation (*comDE*, and *smuT* genes)	[131]

The utilization of sonodynamic therapy (SDT) has been minimally investigated across some studies, employing a range of compounds and ultrasound parameters to target both microbial infections and cancer cells. For example, it has demonstrated significant efficacy in reducing gene expression levels within multispecies bacterial biofilms. This effect is achieved through the generation of ROS and the subsequent downregulation of biofilm-associated genes. Additionally, other natural antimicrobials have exhibited remarkable properties in disrupting cell membrane integrity and impeding protein adhesion, resulting in a reduction in biofilm formation and potent antimicrobial activity. These findings underscore the promising potential of SDT as a versatile therapeutic strategy for combatting microbial infections and cancer, suggesting novel treatments [112,114,115,116].

## 5. Natural Sonophotodynamic Therapy

Some natural compounds have been investigated for their efficacy in sonophotodynamic therapy (SPDT) against microbial infections (Table 4). Resveratrol, sourced from grapes, berries, peanuts, and pines, demonstrated significant antibiofilm properties against different pathogenic bacteria when applied via the aSPDT approach, with a minimum biofilm inhibitory concentration (MBIC) of 512 µg/mL. Furthermore, food colorants such as rhein and E127 cause bacterial inactivation under light or ultrasound exposure (Table 4). The combination of E127 and rhein enhanced these effects, highlighting their potential for antibacterial applications in various industries.

## 6. Approved Preservatives of Natural Origin

There is growing evidence that natural preservatives hold great promise in addressing various industry problems, and their use is increasing worldwide. However, full approval of each preservative is a long-term and puzzling process because of restrictions imposed by regulatory bodies or agencies in every country. This means that preservatives may be put on the “generally recognized as safe (GRAS)” FDA list [134].

There are various lists of approved food preservatives in different countries. For example, since 1962, the European Food Safety Authority (EFSA) has approved the following preservatives of natural origin: benzoic acid and its salts, 4-hydroxybenzoic acid esters, nisin, natamycin, lactic acid, malic acid, and fumaric acid [135]. In addition, essential oils and other natural substances are widespread in the food industry instead of synthetic compounds after the approval by the Food and Drug Administration (FDA) [136].

Interestingly, lactobionic acid seems to be an attractive compound for health care and the food industry because it has antimicrobial, antioxidant, chelating, moisturizing, and gelling properties, but only the US Food and Drug Administration approved it for use in the salt form [137].

The European Food Safety Authority (EU) and FDA approved the extract of *Rosmarinus officinalis* and its compounds as preservatives [138]. The laminaria species *Himanthalia elongata*, *Palmaria palmata,* and *Undaria pinnatifida* seaweeds with antimicrobial properties have been assessed and approved by the European Food Safety Authority (EFSA) [139].

In fact, many natural products have not yet been approved as food and drug preservatives because of the strict requirements for preservatives, rigid regulations, standards, and lengthy toxicological evaluations by the FDA and the European Union.

## 7. Conclusions

To date, an increase in microbial infection has been observed globally. The existing drugs and preservatives may lack effectiveness and safety. Various components from natural sources have attracted researchers to combating this issue and advancing the development of novel natural preservatives. Various plants, animals, and products of animal origin and microorganisms serve as excellent sources for the isolation of active antimicrobial agents. In addition, these agents may be altered or enhanced for use as food preservatives through photodynamic or sonodynamic technologies and delivery techniques such as encapsulation, nanotechnology, and edible packaging.

Future investigations should focus on the quality control of natural preservatives because of inconclusive data on their safety and toxicity. Future research on the influence of antimicrobial agents on different strains and their antibacterial modes of action is needed for progress in this field. In addition, it is essential to find optimal concentrations and combinations of various antimicrobial compounds for food preservation to investigate their possible synergistic effects.

## Figures and Tables

**Table 4:** Natural antimicrobial compounds used in sono-photodynamic therapy (SPDT).

Compounds	Origin	Microorganism	Light and Ultrasound Parameters	Effect	References
Curcumin	*Curcuma longa*	*Listeria monocytogenes*	LED wavelength: 425 nm for 30 min, 800 W ultrasound, curcumin concentration: 3.7 mg/mL for 30 min.	4 log drop in CFU	[125]
		*S. aureus*	Frequency: 1 MHz, pulse repetition frequency: 100 Hz, 20% duty cycle, 3 W/cm^2^, power density: 35–70 J/cm^2^, 15–32 min, UV light: 455 nm	SPDT resulted in a 7.43 log reduction in bacterial inactivation, with a 71% decrease in bacterial adhesion, a 90% reduction in metabolic activity, and reduced biofilm biomass	[122]
		*Acinetobacter baumannii*	Ultrasound power: 28.7–45.2 mW/cm^2^ for 4 min, irradiation ultrasound frequency: 1 MHz, pulse repetition frequency: 100 Hz; wavelength: 450 nm; power intensity: 150 Mw/cm^2^, concentration: 2.5 mg/mL for 5 min.	SDT caused a reduction in and effectively addressed infections caused by *Acinetobacter baumannii* bacteria	[129]
Resveratrol	Grapes, berries, peanuts, pines	*Candida albicans*, *S. aureus*, *S. sobrinus*, and *A. naeslundii*	Concentration: 512 µg/mL, US frequency: 30 kHz with a spatial average ultrasonic intensity of 3 W/cm^2^ for 1 min. Light wavelength: 450 nm with an output intensity of 1000 ± 1400 mW/cm^2^ for 1 min.	The MBIC for Resveratrol was 512 µg/mL. Treatment with aSPDT at this concentration significantly reduced biofilm size and effectively suppressed microbial biofilm growth	[132]
Rhein and E127	Rhubarb, aloe, cascara buckthorn	*S. aureus* and *E. coli*	Ultrasound Frequency: 38 KHz, field strength: 4.1 W/cm^3^ with a sonication time of 10 or 30 sec, LED illumination for 5 or 10 min; light intensity and fluence rate: 137 klux and 1.6 mW/cm^2^ for 30 min.	Bacterial inactivation under light and ultrasound exposure	[133]

## Abbreviations

AMPs	antimicrobial peptides
FDA	Food and Drug Administration
PDT	photodynamic therapy
PSs	photosensitizers
ROS	reactive oxygen species
SDT	sonodynamic therapy
SPDT	sonophotodynamic therapy
